# Altered Spontaneous Brain Activity in Schizophrenia: A Meta-Analysis and a Large-Sample Study

**DOI:** 10.1155/2015/204628

**Published:** 2015-06-09

**Authors:** Yongjie Xu, Chuanjun Zhuo, Wen Qin, Jiajia Zhu, Chunshui Yu

**Affiliations:** ^1^Department of Radiology, Tianjin Key Laboratory of Functional Imaging, Tianjin Medical University General Hospital, No. 154 Anshan Road, Heping District, Tianjin 300052, China; ^2^Tianjin Anning Hospital, Tianjin 300300, China

## Abstract

Altered spontaneous brain activity as measured by ALFF, fALFF, and ReHo has been reported in schizophrenia, but no consensus has been reached on alternations of these indexes in the disorder. We aimed to clarify the regional alterations in ALFF, fALFF, and ReHo in schizophrenia using a meta-analysis and a large-sample validation. A meta-analysis of activation likelihood estimation was conducted based on the abnormal foci of ten studies. A large sample of 86 schizophrenia patients and 89 healthy controls was compared to verify the results of the meta-analysis. Meta-analysis demonstrated that the alternations in ALFF and ReHo had similar distribution in schizophrenia patients. The foci with decreased ALFF/fALFF and ReHo in schizophrenia were mainly located in the somatosensory cortex, posterior parietal cortex, and occipital cortex; however, foci with increased ALFF/fALFF and ReHo were mainly located in the bilateral striatum, medial temporal cortex, and medial prefrontal cortex. The large-sample study showed consistent findings with the meta-analysis. These findings may expound the pathophysiological hypothesis and guide future research.

## 1. Introduction

Schizophrenia is a devastating and disabling neuropsychiatric disorder. The neural mechanisms of this disorder have been attributed to structural and functional abnormalities of the brain [1–5]. Schizophrenia patients have exhibited functional changes in both task-evoked activation and spontaneous brain activity [6, 7]. The spontaneous brain activity can be quantitatively measured by the amplitude of low frequency fluctuations (ALFF), fractional ALFF (fALFF), and regional homogeneity (ReHo) of the blood-oxygen-level-dependent (BOLD) signals derived from resting-state functional magnetic resonance imaging (rs-fMRI) [8, 9].

The ALFF measures the total power of the BOLD signal fluctuations within a specific low frequency range (0.01–0.08 Hz) at the single-voxel level [10]. The fALFF is a normalized index of ALFF, which measures the ratio of the amplitude in a low-frequency band relative to the amplitude in the total frequency bands [11]. The ReHo measures the similarity of the time series of BOLD signals of a given voxel to those of its nearest neighbors in a voxel-wise way that provides important information about the regional temporal synchronization in the brain [12]. Synthetically, the ALFF/fALFF and ReHo provide complementary information about the regional spontaneous brain activity.

The ALFF/fALFF and ReHo have been used to identify functional abnormalities in schizophrenia patients. Nevertheless, studies of ALFF/fALFF and ReHo have yielded inconsistent results [13–22]. For instance, some studies reported that schizophrenia patients had increased ALFF [17, 18], fALFF [13, 18], and ReHo [21, 22] in medial prefrontal cortex (MPFC); however, some others revealed the opposite results [14, 16, 20].

In this study, we combined a meta-analysis and a large-sample study to clarify the two questions: first, regional alterations in ALFF/fALFF and ReHo in schizophrenia; second, the associations in alteration patterns between these measures.

## 2. Materials and Methods

### 2.1. Meta-Analysis

#### 2.1.1. Data Sources and Inclusion Criteria

We reviewed all papers published in PubMed investigating ALFF, fALFF, or ReHo in patients with schizophrenia. The search strategy was carried out with keywords of “schizophrenia” and (“ALFF” or “fALFF” or “ReHo” or “amplitude of low-frequency oscillations” or “amplitude of low frequency fluctuations” or “regional homogeneity”) and (“fMRI” or “functional magnetic resonance imaging”). In order to qualify for inclusion within the meta-analysis, papers were required (a) to report comparisons between schizophrenia patients and matched healthy controls; (b) to employ fMRI; (c) to report results based on voxel-wise analysis; and (d) to detail either the Talairach or Montreal Neurologic Institute (MNI) coordinates of altered brain regions. Studies not fulfilling these requirements were excluded.

Papers were searched independently by two investigators until they made a consensus. After applying the search strategy, we found 20 articles. After carefully reading these articles, we excluded ten of them for the following reasons: (1) three articles were not fMRI studies [23–25]; (2) one article was not focused on schizophrenia patients [26]; (3) three articles were lacking intergroup comparisons [27–29]; (4) one article was focused on imaging and genetic association [30]; and (5) two articles only focused on the ALFF or ReHo changes in the independent components or network nodes [31, 32]. In addition, none of the 10 qualified articles reported negative results. The detailed demographic and clinical data of the 10 qualified articles for meta-analysis are shown in Tables 1 and 2.

#### 2.1.2. Meta-Analysis Procedures

Coordinate-based meta-analysis was performed using the revised version of Activation Likelihood Estimation (ALE) technique [33] implemented in GingerALE 2.3.1 (http://www.brainmap.org/). This algorithm identifies foci showing common activation across different experiments (or studies) if the merged activation is higher than that of the null-distribution reflecting a random spatial association between experiments. Coordinates of the foci reported in the original studies were transformed into the MNI space using the Lancaster transform (icbm2tal tool) in GingerALE. Activation coordinates extracted from each study were weighted to yield estimates of activation likelihood at each voxel, and then a modelled activation (MA) map was computed. The spatial uncertainty of each focus was considered as an independent Gaussian probability distribution. The Gaussian parameters (standard deviation and width) are empirically determined based on between-template and between-subject variances and weighted by the number of subjects. So each voxel has an activation probability value for a specific focus of a certain experiment. Then a MA map for each experiment is computed by summing the probability values for all the foci. After that, the ALE map was calculated by merging all the MA maps of included experiments, which represent the spatial probabilistic distribution about the convergent activation for each voxel. To enable spatial inference on the ALE scores, a nonparametric permutation test was used to generate empirical null-distribution, which reflects the null-hypothesis of a random spatial association between experiments. Finally, each “true” ALE score is then compared to the null-distributed ALE scores to yield a nonparametric *P* value. Because only a few experiments (6 ALFF/fALFF studies and 4 ReHo studies) were enrolled in this meta-analysis, we used an uncorrected intensity threshold of *P* < 0.05 and an extent threshold of 540 mm^3^. The same extent threshold was also applied in the following large-sample study.

### 2.2. Large-Sample Control Study

#### 2.2.1. Participants

A total of 89 schizophrenia patients and 89 healthy controls were recruited in this study. Diagnoses for patients were confirmed using the Structured Clinical Interview for DSM-IV. Exclusion criteria were MRI contraindications, poor image quality, presence of a systemic medical illness or CNS disorder, history of head trauma, and substance abuse within the last 3 months or lifetime history of substance abuse or dependence. Additional exclusion criteria for healthy controls were history of any Axis I or II disorders and first-degree relative with a psychotic disorder. This study was approved by the Medical Research Ethics Committee at Tianjin Medical University General Hospital, and after complete description of the study to the participants, written informed consent was obtained.

#### 2.2.2. Image Data Acquisition

MRI was performed using a 3.0-Tesla MR system (Discovery MR750, General Electric, Milwaukee, WI, USA). Tight but comfortable foam padding was used to minimize head motion, and earplugs were used to reduce scanner noise. Sagittal 3D T1-weighted images were acquired by a brain volume (BRAVO) sequence with the following parameters: repetition time (TR) = 8.2 ms; echo time (TE) = 3.2 ms; inversion time = 450 ms; flip angle (FA) = 12°; field of view (FOV) = 256 mm × 256 mm; matrix = 256 × 256; slice thickness = 1 mm, no gap; and 188 sagittal slices. Resting-state fMRI data were acquired using gradient-echo SENSE-SPIRAL (spiral in) sequence with the following parameters: TR/TE = 1400/30 ms; FOV = 220 mm × 220 mm; matrix = 64 × 64; FA = 60°; slice thickness = 4 mm; gap = 0.5 mm; 32 interleaved transverse slices; 250 volumes. During fMRI scans, all subjects were instructed to keep their eyes closed, to relax and keep motionless, to think of nothing in particular, and not to fall asleep.

#### 2.2.3. ALFF/fALFF Calculation

The resting-state fMRI data were preprocessed as the following steps. The first 10 volumes from each subject were discarded to allow the signal to reach equilibrium and to allow the participant to adapt to the scanning noise. The remaining 240 volumes were corrected for the acquisition time delay between slices. Rigid realignment was then performed to estimate and correct the motion displacement. Three schizophrenia patients were excluded because of excessive head motion; the remaining subjects' fMRI data were within the defined head motion thresholds (translational or rotational motion parameters lower than 2 mm or 2°). Then several nuisance covariates were regressed out from the motion corrected fMRI data, including the mean signals of white matter and cerebrospinal fluid, six rigid motion parameters and their first-level derivatives. We also regressed out spike volumes that had framewise displacement higher than 0.5 to further remove possible influence by head motion [34]. This was realized by generating a nuisance regressor for each time point with “1” for the bad time point and “0” for the remaining time points. A two-step coregistration method was used to transform the regressed fMRI data into the MNI space. First, the mean realigned fMRI images were affinely (12 parameters) coregistered with individual structural images; then the structural images were affinely (12 parameters) coregistered with the standard MNI T1-weighted template. The generated parameters for these two coregistration steps were concatenated and used to normalization of the regressed fMRI data. The normalized fMRI data were resampled into a voxel size of 3 mm × 3 mm × 3 mm. Finally, the normalized fMRI volumes were smoothed with a Gaussian kernel of 6 mm × 6 mm × 6 mm full-width at half maximum (FWHM).

The ALFF was calculated using REST software (http://www.restfmri.net/). The processing procedure was similar to that used in an earlier research [18]. The preprocessed time series were transformed to a frequency domain with a fast Fourier transform (FFT) and the power spectrum was then obtained. Because the power of a given frequency is proportional to the square of the amplitude of this frequency component of the original time series in the time domain, the square root was calculated at each frequency of the power spectrum, and the averaged square root was obtained across 0.01–0.08 Hz at each voxel. This averaged square root was taken as the ALFF. For standardization purposes, the ALFF of each voxel was divided by the global mean ALFF value of a certain subject. The fALFF was calculated based on the method described by Zou et al. [11], which provides a quantitative measure of spontaneous brain activity. In brief, the fALFF was calculated as the ratio of the power spectrum of low frequency (0.01–0.08 Hz) to that of the entire frequency range. For the purpose of standardization, the fALFF value of each voxel was divided by the global mean fALFF value for each subject.

#### 2.2.4. ReHo Calculation

The preprocessing steps for ReHo included slice timing, realignment, regression, band pass filtering (0.01–0.08), and normalization using the same parameters as the ALFF preprocessing. The ReHo was defined as the Kendall correlation coefficient (KCC) of the time series of a given voxel with those of its nearest neighbors (26 voxels) on a voxelwise basis [12]. The KCC can be computed by the following formula:

(1) $$\begin{array}{c} W=\frac{\sum {\left(R_{i}\right)}^{2}-n{\left(\bar{R}\right)}^{2}}{\left(1/12\right)K^{2}\left(n^{3}-n\right)}, \end{array}$$

where *W* is the KCC among given voxels, ranging from 0 to 1; *R*_*i*_ is the sum rank of the *i*th time point; $\bar{R}=[(n+1)K]/2$ is the mean of the *R*_*i*_; *K* is the number of time series within a measured cluster (*K* = 27; one given voxel plus its 26 neighbors); and *n* is the number of ranks (*n* = 240).

#### 2.2.5. Group Statistical Analysis

To explore the ALFF and ReHo differences between the two groups, a two-sample *t*-test was performed on the normalized ALFF/fALFF and ReHo maps in a voxelwise manner. False discovery rate (FDR) correction was applied with a corrected threshold of *P* < 0.05 (two-tailed) and a cluster size of >540 mm^3^. All coordinates were reported in MNI space. Brain regions with significant intergroup differences in ALFF/fALFF/ReHo in the meta-analysis were defined as regions of interest (ROIs). We extracted the ALFF/fALFF/ReHo values of these ROIs from each subject of our own sample and compared them between schizophrenia patients and healthy controls (*P* < 0.05, uncorrected).

## 3. Results

### 3.1. Meta-Analysis

#### 3.1.1. ALFF/fALFF

**Figure 1:**
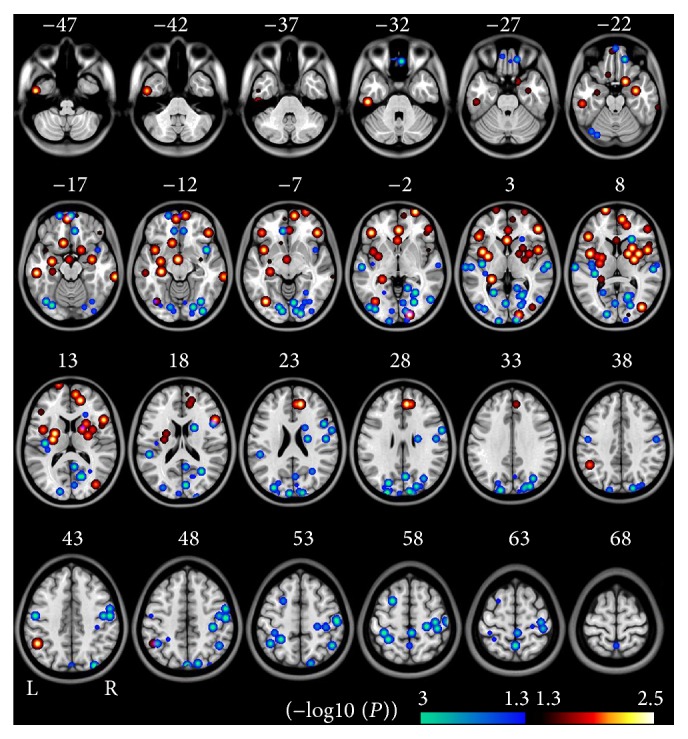
Brain regions with significant differences in ALFF/fALFF in meta-analysis between schizophrenia and healthy controls (*P* < 0.05, uncorrected, cluster size >540 mm^3^). The hot color represents the higher ALFF/fALFF in schizophrenia patients. The cold color represents the higher ALFF/fALFF in healthy controls. The overlapping area is marked in the pink color. Here, it represents the contradictory results between studies. The (−log10 (*P*)) value means the negative ten-logarithm transformation of *P* value. A larger value of (−log10 (*P*))represents a smaller *P* value.

A total of 6 experiments that involved 514 schizophrenia patients and 518 healthy subjects were recruited for this meta-analysis. All the 6 experiments showed both decreased and increased ALFF/fALFF in schizophrenia patients (Table 2). Compared to healthy controls, schizophrenia patients exhibited decreased ALFF/fALFF mainly in the bilateral occipital (OC), sensorimotor (SMC), and posterior parietal cortices (PPC). These patients also had decreased ALFF/fALFF in the bilateral superior temporal gyrus (STG), insula, and medial orbitofrontal cortex (MOFC) (*P* < 0.05, uncorrected). Schizophrenia patients had increased ALFF/fALFF in the bilateral medial (MPFC) and lateral prefrontal cortex (LPFC), medial temporal cortex (MTL), and striatum, and there were scattered foci in the bilateral OC, insula, and PPC (Figure 1 and Table S1 in Supplementary material available online at http://dx.doi.org/10.1155/2015/204628).

#### 3.1.2. ReHo

**Figure 2:**
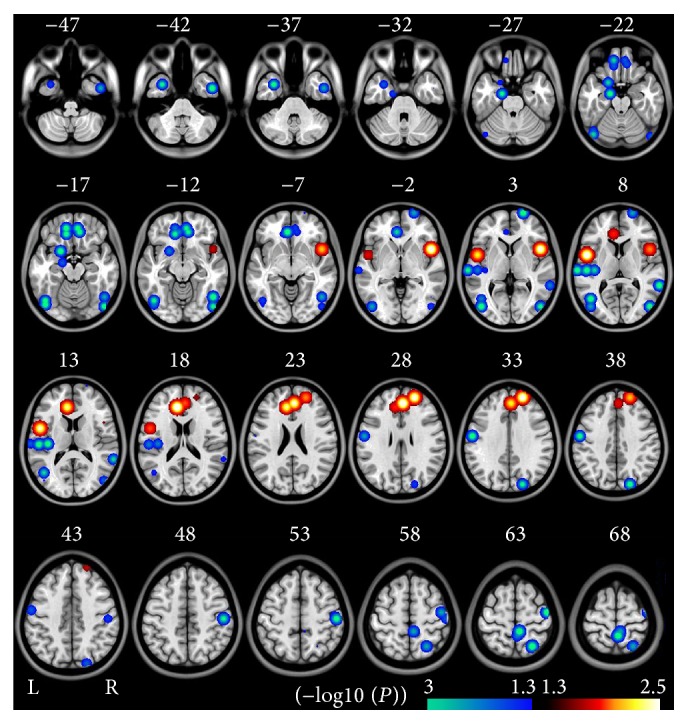
Brain regions with significant differences in ReHo in meta-analysis between schizophrenia and healthy controls (*P* < 0.05, uncorrected, cluster size >540 mm^3^). The hot color represents the higher ReHo in schizophrenia patients. The cold color represents the higher ReHo in healthy controls. The (−log10 (*P*)) value means the negative ten-logarithm transformation of* P* value. A larger value of (−log10 (*P*)) represents a smaller* P* value.

A total of 4 experiments with 141 schizophrenia patients and 142 healthy subjects were recruited for this meta-analysis. All the 4 experiments showed decreased ReHo and only 2 experiments showed increased ReHo in schizophrenia patients (Table 2). Compared to healthy controls, schizophrenia patients showed decreased ReHo mainly in the bilateral MOFC, STG, and OC, and there are scattered foci including bilateral SMC, PPC, and MTL. Schizophrenia patients had a higher ReHo in bilateral MPFC, LPFC, and right insula (*P* < 0.05, uncorrected) (Figure 2 and Table S2).

### 3.2. Validation Study

A total of 86 schizophrenia patients and 89 healthy controls were included in this study. The demographic and clinical data of subjects are shown in Table 3.

#### 3.2.1. ALFF

**Figure 3:**
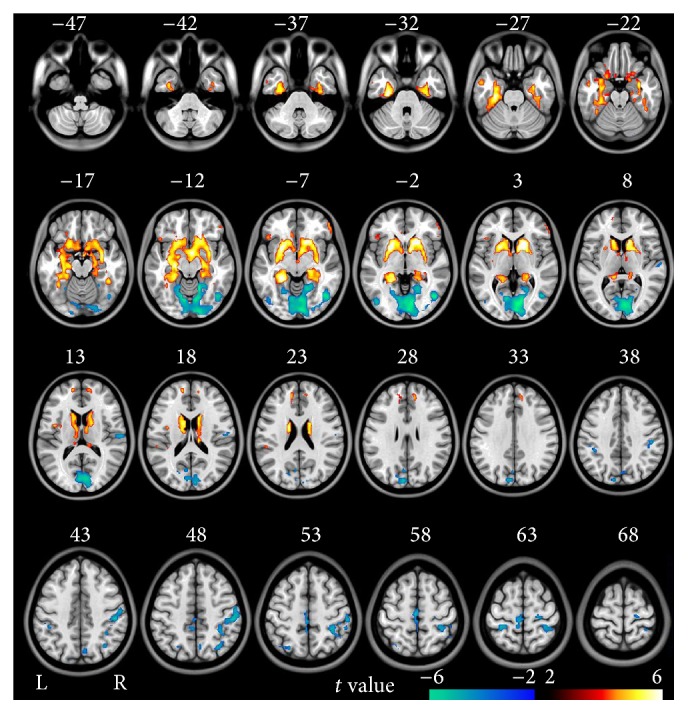
Brain regions with significant differences in ALFF in validation study between schizophrenia patients and healthy controls (FDR < 0.05, two-tailed, cluster size >540 mm^3^). The hot color represents the higher ALFF in schizophrenia patients. The cold color represents the higher ALFF in healthy controls.

In the voxel-based analysis, compared to healthy controls, schizophrenia patients showed decreased ALFF in the bilateral OC, PPC, and SMC and right STG and increased ALFF in bilateral striatum, MTL, MPFC, and lateral orbitofrontal cortex (LOFC) (*P* < 0.05, FDR corrected) (Figure 3).

#### 3.2.2. fALFF

**Figure 4:**
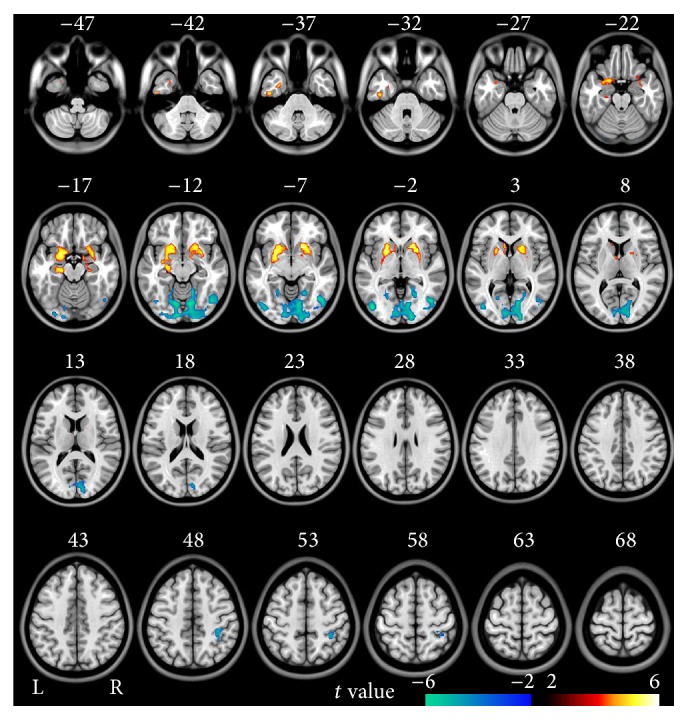
Brain regions with significant differences in fALFF in validation study between schizophrenia patients and healthy controls (FDR < 0.05, two-tailed, cluster size >540 mm^3^). The hot color represents the higher fALFF in schizophrenia patients. The cold color represents the higher fALFF in healthy controls.

In the voxel-based analysis, compared to healthy controls, schizophrenia patients showed decreased fALFF mainly in bilateral OC and right postcentral gyrus and increased fALFF in bilateral striatum and MTL (*P* < 0.05, FDR corrected) (Figure 4).

#### 3.2.3. ReHo

**Figure 5:**
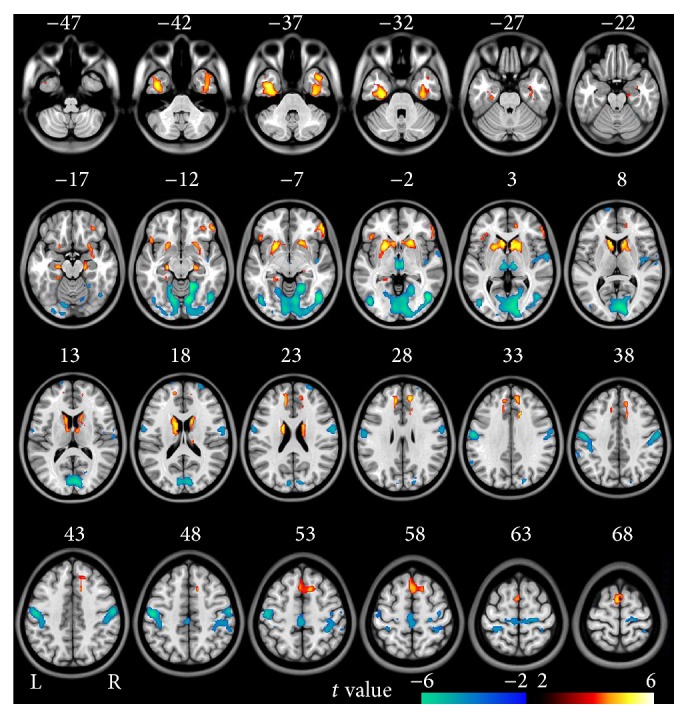
Brain regions with significant differences in ReHo in validation study between schizophrenia patients and healthy controls (FDR < 0.05, two-tailed, cluster size >540 mm^3^). The hot color represents the higher ReHo in schizophrenia patients. The cold color represents the higher ReHo in healthy controls.

In the voxel-based analysis, compared to healthy controls, schizophrenia patients showed decreased ReHo in bilateral OC, SMC, thalamus, frontal pole, and right STG. Schizophrenia patients also showed increased ReHo in bilateral striatum, MTL, LOFC, MPFC, and SMA (*P* < 0.05, FDR corrected) (Figure 5).

#### 3.2.4. ROI-Based Validation

The results of ROI-based intergroup comparisons in ALFF/fALFF/ReHo are shown in Tables S1 and S2. We found that more than a half of ROIs with significant intergroup differences in ALFF/fALFF/ReHo in the meta-analysis also had significant intergroup differences in our sample (*P* < 0.05, uncorrected).

## 4. Discussion

After the first reports of ALFF [14] and ReHo [19] abnormalities in schizophrenia, several studies were conducted to investigate the altered ALFF and ReHo in this disorder, but the results were inconsistent. The reasons for these inconsistent results were complicated, and it was necessary to reconcile these conflicting results. Therefore, we combined a meta-analysis and a large-sample study to clarify the regional alterations of ALFF and ReHo in schizophrenia. We demonstrated that both ALFF/fALFF and ReHo were decreased in the bilateral OC, SMC, and PPC and increased in the bilateral striatum, MTL, and MPFC in schizophrenia patients.

### 4.1. Foci with Consistently Decreased ALFF/ReHo in Schizophrenia

The reduced ALFF and ReHo of the occipital cortex in schizophrenia patients were observed in most of studies, which are consistent with deficits in low level visual processing in schizophrenia [35–38]. The functional abnormality of the occipital cortex has also been related to visual hallucinations and object-recognition deficits in schizophrenia [39, 40]. Moreover, Schechter et al. suggested that schizophrenia patients took 75% greater time in processing magnocellular-aimed stimuli and 20% longer duration in detecting parvocellular-aimed stimuli in the visual backwards masking experiment than healthy controls [41].

The PPC plays a key role in high-level cognitive processing and the precuneus is an important node of the default-mode network (DMN). The reduced ALFF/ReHo in the PPC may be consistent with the notion that the lower ALFF and ReHo of the PPC predict worse performance in working memory [42], whose function has been found to be impaired in schizophrenia [43]. The reduced ALFF/ReHo in the precuneus is also in agreement with the structural and functional deficits in this region in patients with schizophrenia [44–46].

We also observed decreased ALFF/fALFF and ReHo in the sensorimotor cortex (SMC) in schizophrenia, which is consistent with previous finding of the grey matter abnormalities in this region in schizophrenia [47, 48]. The structural and functional impairment in the SMC in schizophrenia may be related to the increased involuntary movements in schizophrenia [49]. The SMC impairment may be also associated with neurological soft signs, describing the neurological abnormalities in sensory integration, motor regulation, sequencing complex motor acts, and primitive reflexes that occur in the majority of schizophrenia patients [50].

### 4.2. Foci with Consistently Increased ALFF/ReHo in Schizophrenia

We observed increased ALFF and ReHo in the striatum in schizophrenia, which are consistent with increased cerebral blood flow (CBF) and glucose metabolism in medicated patients with schizophrenia [51]. In drug-naïve schizophrenia patients, the striatum exhibited decreased volume and CBF compared to healthy controls [52, 53]. Thus, the increased spontaneous activity in the striatum may reflect the effects of antipsychotic drugs. The involvement of the striatum in schizophrenia is also supported by the dopamine hypothesis of schizophrenia, which postulates hyperdopaminergia in the striatum [54]. Indeed, increased striatal dopamine transmission has been reported in first-episode schizophrenia patients [55, 56].

We also found increased ALFF and ReHo in the hippocampus in schizophrenia, which are well consistent with increased cerebral blood flow (CBF) and glucose metabolism in this region in patients with schizophrenia [57]. The hippocampal disconnection has also been found in schizophrenia [58, 59], which may be associated with cognitive and emotional dysfunction.

### 4.3. Foci with Inconsistent Reports in the Schizophrenia

The OFC and MPFC are critical for social-emotional and insight processing. The impairment of these regions has been associated with emotionally instable, irritable, impulsive, and loss of insight in schizophrenia and other disorders [60–62]. Inconsistent alterations in ALFF and ReHo have been reported in the MPFC and OFC in schizophrenia patients, either decrease [14–16, 19, 20] or increase [13, 17, 18, 21, 22]. Our data only demonstrated increased ALFF and ReHo in the MPFC and OFC. Although the age, sex, illness duration, and medication may be related to the discrepancy, imaging sequence may also play a role. All previous studies on ALFF or ReHo used a single-shot echo planar imaging (EPI) sequence to collect the data. This sequence can inevitably induce image distortion and signal loss in the OFC due to phase error accumulation and susceptibility. In contrast, we applied a SENSE-SPIRAL sequence to reduce the above-mentioned artifacts, which may help us to obtain more plausible results.

The insula has demonstrated structural atrophy in schizophrenia [63–67]. Recent studies have revealed the functional disconnection of the insula in this disorder [65, 66, 68–72]. However, either increased [17, 18, 22] or decreased [13, 16, 18, 22] ALFF/ReHo has been reported in the insula in schizophrenia. Although our results did not show ALFF/ReHo changes in the insula in schizophrenia, the meta-analysis showed that the increased ALFF/ReHo was mainly located in the anterior insula, whereas the decreased ALFF/ReHo was mainly located in the posterior insula. Recent studies have shown the different insula subregions demonstrated diverse connectivity patterns and functions: the anterior insula is closely connected with limbic system, middle and inferior temporal cortex, and anterior cingulate cortex, which plays a role in processing of emotion, attention, and salience; the posterior part is closely connected with the premotor, sensorimotor, supplementary motor, and middle-posterior cingulate cortices, which is related with sensorimotor integration [73, 74]. So the different change patterns in the anterior and posterior parts of the insula may reflect different aspect symptoms in the schizophrenia. Actually, the functional disconnection of the anterior insula has been associated with cognitive deficit in patients with schizophrenia [68].

Although the alternations of ALFF and ReHo showed similar distribution in schizophrenia patients, they also have some differences. On one hand, these two indices both reflect the spontaneous neural activity. Strong positive correlation has been shown between the two indices [75], and both ALFF and ReHo were found to correlate with cerebral blood flow [76]. The close association between ALFF and ReHo can interpret the consistent findings in schizophrenia. On the other hand, these two indices reflect different aspects of the spontaneous neural activity. The ALFF measures the low-frequency spontaneous fluctuation of neural activity for a certain voxel, while ReHo measures the regional homogeneity of spontaneous neural activity among neighboring voxels, which may interpret the differential findings in ALFF and ReHo. Brain regions with both ALFF and ReHo changes may enhance our confidence to conclude that these brain regions have altered spontaneous brain activity in schizophrenia. Some brain regions that only showed changes in ALFF or in ReHo indicate that the ALFF and ReHo can provide complementary information about the regional spontaneous brain activity.

Several limitations should be noted when one interpreting our findings. The reliability of an ALE analysis depends on the number of studies included. Too few studies may result in separate small foci (like in this study), which could be improved only when a large number of studies were included. A limitation of ALE is that the negative results cannot be included in the analysis; however, none of the qualified studies reported negative results which may lower the effect. The demographic characteristics are different across studies, particularly in symptoms, illness duration, and medication. Thus our results may reflect the generalized changes in spontaneous neural activity in a mixed sample of schizophrenia patients. It has been suggested that eyes-states (open or close) may affect the spontaneous brain activity [77]; however, at least 8/10 studies (the remaining 2 studies did not mention eyes-states) adopted an eye-closed scheme. Thus our results are more likely a reflection of eye-closed state. When normalization is done, it may also influence our results. However, all the 10 studies included in the meta-analysis performed normalization prior to obtaining derived connectivity metrics. In order to keep pace with the meta-analysis, we also performed normalization prior to obtaining derived connectivity metrics in the large-sample study. In our meta-analysis, a loose threshold (*P* < 0.05, uncorrected) was used, which may prevent us from drawing a strong conclusion. However, we think our meta-analysis in combination with a large-sample study may provide a more comprehensive understanding of the change patterns of brain spontaneous activity in schizophrenia.

## 5. Conclusions

We performed a meta-analysis and a large-sample study on the alternations in ALFF and ReHo in schizophrenia. Our findings suggest that schizophrenia patients demonstrate an increased spontaneous brain activity in the striatum, medial temporal cortex, and medial prefrontal cortex and a decreased activity in the sensorimotor cortex, posterior parietal cortex, and occipital cortex. These findings may help to expound the pathophysiological hypothesis and to guide future researches.

## Supplementary Material

The coordinates of the meta-analysis and the results of ROI-based intergroup comparisons in ALFF/fALFF/ReHo are shown in Tables S1 and S2. We found that more than a half of ROIs with significant intergroup differences in ALFF/fALFF/ReHo in the meta-analysis also had significant intergroup differences in our sample (*P* < 0.05, uncorrected).

## Figures and Tables

**Table 1:** The demographic and clinical data of studies included in the meta-analysis.

References	Publication year	Scanners	Indices	Patients age (years)	Controls age (years)	Patients (cases/F)	Controls (cases/F)	Diagnosis	Duration of illness	PANSS total	PANSS positive	PANSS negative
Hoptman et al. [13]	2010	Siemens 1.5T	ALFF/fALFF	36.5 ± 11.0	41.9 ± 10.9	29/3	26/7	CS	13.0 ± 7.2 (years)	76.5 ± 16.6	18.4 ± 6.2	20.2 ± 6.2
Huang et al. [14]	2010	GE 3T	ALFF	24.2 ± 8.4	24.5 ± 8.6	66/36	66/36	FES	8.8 ± 14.1 (months)	107.2 ± 15.1	26.4 ± 5.2	20.7 ± 6.3
He et al. [15]	2013	GE 3T	fALFF	25.4 ± 8.3	26.6 ± 8.9	104/55	104/54	FES	39.5 ± 31.9 (weeks)	92.1 ± 17.5	—	—
Ren et al. [16]	2013	GE 3T	ALFF	24.3 ± 7.5	24.4 ± 7.6	100/59	100/59	FES	6.3 ± 11.0 (months)	97.9 ± 17.8	25.1 ± 6.0	18.8 ± 7.7
Turner et al. [17]^*∗*^	2013	Siemens/GE 3T	ALFF/fALFF	34.4–44.8	34.4–42.5	146/35	160/46	CS	13.1–24.1 (years)	53.8–64.7	13.5–16.5	13.1–16.7
Yu et al. [18]	2014	Siemens 3T	ALFF/fALFF	31.7 ± 9.6	29.9 ± 8.6	69/35	62/27	CS	7.1 ± 6.5 (years)	52.9 ± 16.8	12.1 ± 4.7	13.4 ± 6.1
Liu et al. [19]	2006	GE 1.5T	ReHo	23.7 ± 4.4	24.4 ± 3.9	18/9	18/9	Mixed	26.8 ± 19.2 (months)	80.4 ± 18.7	—	—
Jiang et al. [20]	2010	GE 1.5T	ReHo	16.4 ± 0.8	16.3 ± 0.9	18/9	18/9	EOS	9.6 ± 5.9 (months)	91.8 ± 6.7	—	—
Chen et al. [21]	2013	Siemens 3T	ReHo	28.3 ± 1.43	35.7 ± 1.8	36/20	44/27	FES	—	25.2 ± 6.9	—	—
Yu et al. [22]	2013	Siemens 3T	ReHo	31.7 ± 9.6	29.9 ± 8.6	69/36	62/27	CS	7.1 ± 6.5 (years)	52.9 ± 16.8	12.1 ± 4.7	13.4 ± 6.1

^*∗*^Turner et al. [17] present the demographic information in 7 sites, respectively. Thus we only show the ranges of mean values from the 7 sites.

All studies did not give the medication information except for the study of Hoptman et al. [13], so we did not list the information in the table.

F, female; PANSS, Positive and Negative Syndrome Scale; FES, first-episode schizophrenia; CS, chronic schizophrenia; Mixed, FES and CS; EOS, early onset schizophrenia.

**Table 2:** Detailed area of studies included in the meta-analysis.

Regions	ALFF		fALFF		ReHo	
	Increase	Decrease	Increase	Decrease	Increase	Decrease
L-MPFC		[14, 16]			[22]	[20]
R-MPFC	[17, 18]		[13, 18]		[21, 22]	[20]
OFC	[17]	[16]	[13]	[15]		[19]
R-PCL		[17]				[19]
L-post_CG						[19]
R-post_CG		[13, 17, 18]				[19, 22]
L-preCG		[18]		[18]		[22]
R-preCG		[13, 17, 18]		[18]		[22]
L-STG		[17]				[19, 21]
R-STG		[17]				[19]
L-MTG	[18]					[19]
R-MTG	[18]			[17]		
L-ITG	[17]					[19]
R-ITG						[19]
L-HG				[13]		[22]
L-PH	[13]					[19]
R-PH		[18]	[13]			
L-Fus	[17]			[18]		
L-HP	[17]			[13]		
L-aINS	[17]		[18]		[22]	
R-aINS		[16]	[18]		[22]	
L-pINS	[17]	[18]		[13]		[22]
L-MOG		[13, 18]		[13, 18]		[19, 22]
R-MOG	[16]	[13]		[13]		[19]
L-IOG	[16]					[19]
R-IOG				[18]		[19]
L-Cun		[13]		[13]		
R-Cun		[13, 17]		[13, 17, 18]		
L-LG		[13, 18]				
R-LG		[13, 18]		[13, 18]		
R-Cal	[16]					
L-Cau			[13]			
R-Cau				[13]		
L-Put	[14, 16]		[15]			
R-Put	[14, 16, 17]		[15]			
L-HTh	[17]					
L-Cla				[13]		
R-LN			[13]			
L-ACC						[19]
R-PCC		[17]		[13, 17]		
R-SPL						[19]
L-IPL		[16, 18]	[18]	[18]		
R-IPL		[16]				
L-Pcu		[13]				
R-Pcu		[13, 16–18]		[13]		[19, 22]

L, left; R, right; MPFC, medial prefrontal cortex; PCL, paracentral lobule; post_CG, postcentral gyrus; preCG, precentral gyrus; STG, superior temporal gyrus; MTG, middle temporal gyrus; ITG, inferior temporal gyrus; HG, Heschl gyrus; PH, parahippocampal; Fus, fusiform; HP, hippocampus; aINS, anterior insula; pINS, posterior insula; MOG, middle occipital gyrus; IOG, inferior occipital gyrus; Cun, cuneus; LG, lingual gyrus; Cal, calcarine; Cau, caudate; Put, putamen; HTh, hypothalamus; Cla, claustrum; LN, lentiform nucleus; ACC, anterior cingulate cortex; PCC, posterior cingulate cortex; SPL, superior parietal lobule; IPL, inferior parietal lobule; Pcu, precuneus.

**Table 3:** Demographic and clinical data of participants in the large-sample study.

Variables	Patients (*N* = 86)	Controls (*N* = 89)	*P*
Age (years)	33.4 ± 7.8	33.5 ± 10.6	0.957
Gender (males/females)	46/40	40/49	0.258
Illness duration (months)	16.3 ± 41.1	—	—
PANSS total	70.5 ± 23.4	—	—
PANSS positive	16.6 ± 8.1	—	—
PANSS negative	20.2 ± 8.9	—	—

Among the 86 schizophrenia patients, 6 patients were first-episode and 80 patients were chronic.
