# Supplementary material for: Altered Spontaneous Brain Activity in Schizophrenia: A Meta-Analysis and a Large-Sample Study
Source: Biomed Res Int. 2015 Jun 9;2015:204628. doi: 10.1155/2015/204628 (PMC4477065; doi:10.1155/2015/204628)
Supplement: Supplementary file 1 — The coordinates of the meta-analysis and the results of ROI-based intergroup comparisons in ALFF/fALFF/ReHo are shown in Tables S1 and S2. We found that more than a half of ROIs with significant intergroup differences in ALFF/fALFF/ReHo in the meta-analysis also had significant intergroup differences in our sample (P < 0.05, uncorrected). [file 204628.f1.pdf]

Supplementary Materials

The coordinates of the meta-analysis and the results of ROI-based intergroup comparisons in ALFF/fALFF/ReHo are shown in Tables S1 and S2. We found that more than a half of ROIs with significant intergroup differences in ALFF/fALFF/ReHo in the meta-analysis also had significant intergroup differences in our sample ($P < 0.05$, uncorrected).

Table S1. The differences in ALFF/fALFF between schizophrenia patients and healthy controls in meta-analysis and ROIs analysis.

Regions	Meta-analysis					ROIs analysis		
	Voxels	MNI			Peak Intensity [1]	Patients (mean ± SD)	Controls (mean ± SD)	P
		X	Y	Z				
Increased in SCZ								
***L-ITG**	167	-52	-6	-46	3.43	0.53 ± 0.19	0.48 ± 0.18	0.051
**L-ITG/MTG**	472	-54	-28	-18	3.72	0.90 ± 0.20	0.85 ± 0.18	0.069
***R-HP**	320	32	-6	-20	3.72	0.82 ± 0.13	0.76 ± 0.11	0.001
***R-OL**	250	16	8	-22	3.43	0.70 ± 0.17	0.62 ± 0.13	0.001
***R-MTG**	191	68	-34	-16	3.22	1.10 ± 0.31	1.07 ± 0.33	0.464
**L-HP**	936	-30	-10	-16	3.43	0.72 ± 0.08	0.64 ± 0.07	0.001
***L-Rec**	320	-12	20	-16	3.72	0.68 ± 0.13	0.61 ± 0.11	0.001
**L-OFC**	624	-6	58	-16	3.72	1.33 ± 0.29	1.28 ± 0.29	0.231
***L-HP**	315	-4	-6	-12	3.72	1.20 ± 0.39	0.97 ± 0.20	0.001
**L-INS/Put**	1545	-28	-12	12	3.72	0.79 ± 0.08	0.75 ± 0.08	0.001
**R-IFG**	618	48	42	2	3.72	1.30 ± 0.23	1.22 ± 0.21	0.014
***L-IOG**	341	-36	-72	-6	5.06	0.76 ± 0.15	0.80 ± 0.16	0.048
**L-IFG**	591	-42	36	-4	3.72	1.28 ± 0.23	1.24 ± 0.23	0.171
**Bi_ACC**	639	0	40	-2	3.72	1.04 ± 0.16	1.02 ± 0.19	0.443
***R-SFG**	161	26	68	-4	3.22	1.26 ± 0.36	1.20 ± 0.39	0.207
***R-Cal**	320	18	-94	0	3.72	1.01 ± 0.31	1.27 ± 0.39	0.001
**R-Put/INS**	1731	48	8	6	3.72	0.88 ± 0.08	0.85 ± 0.09	0.051
***R-MOG**	274	38	-82	8	3.22	1.00 ± 0.30	1.04 ± 0.20	0.230
**R-ACC/SFG**	1095	12	42	24	5.06	1.11 ± 0.16	1.05 ± 0.17	0.007
***L-SFG**	156	-18	72	8	3.72	0.99 ± 0.30	1.10 ± 0.29	0.021
***L-IPL**	320	-48	-52	42	3.72	1.57 ± 0.35	1.69 ± 0.31	0.010
Decreased in SCZ								
**R-SFG**	326	14	44	-24	3.08	0.84 ± 0.27	0.83 ± 0.36	0.752
**L-Fus**	314	-30	-78	-18	4.36	0.89 ± 0.24	0.92 ± 0.25	0.190
***L-Rec**	140	0	62	-18	3.48	1.26 ± 0.35	1.23 ± 0.37	0.488
***L-SFG**	144	-18	62	-16	3.08	0.59 ± 0.24	0.54 ± 0.19	0.119

***R-IOG**	148	36	-88	-12	3.48	0.87 ±0.29	1.00 ±0.27	0.001
**R-LG/Cal**	1824	8	-74	-6	4.36	0.96 ±0.18	1.14 ±0.24	0.001
***R-INS**	150	42	8	-12	3.48	1.37 ±0.20	1.34 ±0.25	0.408
**L-LG**	280	-10	-88	-8	3.08	0.96 ±0.30	1.19 ±0.43	0.001
***L-OFC**	160	-10	38	-10	2.96	0.75 ±0.18	0.71 ±0.17	0.095
**L-MOG**	285	-36	-84	0	4.36	0.89 ±0.20	0.97 ±0.21	0.006
***R-MTG**	139	50	-70	0	3.08	1.06 ±0.27	1.24 ±0.40	0.001
**L-STG**	290	-66	-18	6	4.36	1.16 ±0.24	1.15 ±0.24	0.938
**L-MOG**	280	-16	-92	10	4.36	0.91 ±0.25	1.01 ±0.26	0.012
***L-Cal**	171	-22	-58	4	4.36	0.90 ±0.22	0.98 ±0.30	0.032
**R-STG**	290	56	-22	6	3.08	1.41 ±0.28	1.50 ±0.30	0.011
**L-INS**	428	-40	-14	4	3.48	0.93 ±0.18	0.87 ±0.13	0.014
***L-LG**	160	-4	-76	4	2.96	1.32 ±0.42	1.65 ±0.57	0.001
***R-Cau**	150	22	26	8	3.48	0.59 ±0.08	0.56 ±0.10	0.016
***R-Pcu**	150	6	-54	14	3.48	1.68 ±0.36	1.73 ±0.38	0.543
**R-SOG/Cun**	784	6	-82	16	3.48	1.25 ±0.27	1.38 ±0.29	0.001
***R-Pcu**	152	30	-62	18	3.08	0.73 ±0.15	0.71 ±0.13	0.298
***L-STG**	150	-50	-36	20	3.48	1.11 ±0.32	0.99 ±0.21	0.002
***R-Cau**	152	18	6	18	3.08	0.66 ±0.12	0.60 ±0.10	0.001
**L-SOG**	450	-18	-90	34	4.36	1.09 ±0.29	1.15 ±0.24	0.127
***R-IFG**	150	48	12	20	3.48	1.23 ±0.25	1.31 ±0.25	0.034
***L-Cun**	104	-6	-96	22	2.96	0.95 ±0.31	1.04 ±0.29	0.061
***R-Cau**	160	22	-12	24	2.96	0.60 ±0.95	0.54 ±0.08	0.001
***R-post_CG**	160	50	-12	24	2.95	0.99 ±0.22	1.08 ±0.27	0.020
***R-preCG**	160	60	2	26	4.36	0.99 ±0.26	1.09 ±0.35	0.027
***R-Pcu**	152	4	-70	26	3.08	1.97 ±0.50	2.18 ±0.45	0.004
***L-post_CG**	150	-52	-12	42	3.48	1.02 ±0.29	1.11 ±0.25	0.032
**R-preCG**	554	54	-12	42	4.36	0.99 ±0.22	1.08 ±0.21	0.004
***R-SPL**	109	14	-80	50	3.08	1.05 ±0.10	1.13 ±0.11	0.001
**R-post_CG**	581	36	-28	48	3.48	0.86 ±0.23	0.96 ±0.24	0.004
***R-IPL**	122	44	-54	50	3.08	1.78 ±0.44	2.03 ±0.60	0.001
***L-IPL**	152	-42	-52	50	3.08	1.59 ±0.37	1.64 ±0.33	0.148
**L- post_CG**	364	-36	-36	56	3.48	0.91 ±0.19	0.98 ±0.21	0.014
***L-MFG**	152	-24	8	56	3.08	1.14 ±0.25	1.18 ±0.25	0.302
***R- PCL**	150	2	-36	60	3.48	0.93 ±0.21	1.05 ±0.28	0.003
***L-Pcu**	140	0	-54	62	3.48	1.67 ±0.48	1.69 ±0.35	0.789

1 Peak Intensity is the value of (-log10(P)).

* Those results composed of only one circle respectively.

Abbreviations: L, left; R, right; Bi, bilateral; ITG, inferior temporal gyrus; MTG, middle temporal gyrus; HP, hippocampus; OL, olfactory lobe; Rec, rectus; OFC, orbitofrontal cortex; INS, insula; Put, putamen; IFG, inferior frontal gyrus; IOG, inferior occipital gyrus; ACC, anterior cingulate cortex; SFG, superior frontal gyrus; Cal, calcarine; MOG, middle occipital gyrus; IPL, inferior parietal lobule; Fus, fusiform; LG, lingual gyrus; STG, superior temporal gyrus; SOG, superior occipital gyrus; Cun, cuneus; Cau, caudate; preCG, precentral gyrus; post_CG, postcentral gyrus;

Pcu, precuneus; SPL, superior parietal lobule; MFG, middle frontal gyrus; PCL, paracentral lobule.

Table S2. The differences in ReHo between schizophrenia patients and healthy controls in meta-analysis and ROIs analysis.

Regions	Meta-analysis					ROIs analysis		
	Voxels	MNI			Peak Intensity [1]	Patients (mean ± SD)	Controls (mean ± SD)	P
		X	Y	Z				
Increased in SCZ								
***R-INS**	1498	48	12	0	5.36	1.03 ±0.09	1.03 ±0.10	0.772
***L-IFG**	1472	-52	2	8	3.40	0.94 ±0.07	0.95 ±0.09	0.282
**L-ACC**	3681	-10	36	18	4.88	1.13 ±0.08	1.10 ±0.08	0.019
Decreased in SCZ								
***R-ITG**	442	48	0	-42	5.06	0.68 ±0.13	0.63 ±0.11	0.008
***L-ITG**	531	-34	6	-40	3.59	0.65 ±0.10	0.61 ±0.08	0.001
**L-PH/L-HP**	1003	-18	-10	-24	4.02	0.80 ±0.09	0.76 ±0.07	0.001
**L-MOG/IOG**	1546	-46	-76	-18	3.59	1.04 ±0.10	1.07 ±0.10	0.008
**L-Rec/ACC**	1933	-12	44	-18	4.02	0.93 ±0.15	0.93 ±0.16	0.872
**R-ITG/ IOG**	694	48	-78	-16	4.02	0.98 ±0.12	1.06 ±0.13	0.001
***R-SFG**	341	20	68	2	3.28	1.03 ±0.19	1.03 ±0.16	0.706
**R-MOG**	264	48	-78	6	5.06	1.09 ±0.15	1.15 ±0.15	0.002
**L-STG**	1413	-66	-22	6	4.02	0.90 ±0.08	0.92 ±0.07	0.091
***R-MTG**	376	62	-46	8	3.28	1.15 ±0.15	1.11 ±0.14	0.082
***L-post_CG**	577	-58	-10	36	3.59	0.96 ±0.14	1.04 ±0.14	0.001
**R-SOG**	402	20	-84	36	4.02	1.09 ±0.14	1.15 ±0.15	0.011
**R-post_CG**	547	44	-12	62	3.59	1.11 ±0.16	1.14 ±0.16	0.001
***R-SPL**	390	24	-60	62	4.02	1.14 ±0.16	1.15 ±0.13	0.336
**R-PCL/Pcu**	741	6	-40	62	3.59	0.94 ±0.12	0.98 ±0.14	0.126

1 Peak Intensity is the value of (-log10(P)).

* Those results composed of only one circle respectively.

Abbreviations: L, left; R, right; INS, insula; IFG, inferior frontal gyrus; ACC, anterior cingulate cortex; ITG, inferior temporal gyrus; PH, paraHippocampus; HP, hippocampus; MOG, middle occipital gyrus; MTG, middle temporal gyrus; IOG, inferior occipital gyrus; Rec, rectus; SFG, superior frontal gyrus; STG, superior temporal gyrus; SOG, superior occipital gyrus; post_CG, postcentral gyrus; preCG, precentral gyrus; SPL, superior parietal lobule; PCL, paracentral lobule; Pcu, precuneus.
